# Deep learning-based acceleration of muscle water T2 mapping in patients with neuromuscular diseases by more than 50% - translating quantitative MRI from research to clinical routine

**DOI:** 10.1371/journal.pone.0318599

**Published:** 2025-04-16

**Authors:** Joachim Schmitt, Dominik Weidlich, Kilian Weiss, Jonathan Stelter, Federica Montagnese, Marcus Deschauer, Benedikt Schoser, Claus Zimmer, Dimitrios C. Karampinos, Jan S. Kirschke, Sarah Schlaeger

**Affiliations:** 1 Department of Diagnostic and Interventional Neuroradiology, School of Medicine and Health, TUM Klinikum Rechts der Isar, Technical University of Munich, Munich, Germany; 2 Department of Diagnostic and Interventional Radiology, School of Medicine and Health, TUM Klinikum Rechts der Isar, Technical University of Munich, Munich, Germany; 3 Philips GmbH, Hamburg, Germany; 4 Department of Neurology, Friedrich-Baur-Institute, LMU Munich, Munich, Germany; 5 Department of Neurology, School of Medicine and Health, TUM Klinikum Rechts der Isar, Technical University of Munich, Munich, Germany; 6 Department of Radiology, LMU University Hospital, LMU Munich, Munich, Germany.; Memorial Sloan Kettering Cancer Center, UNITED STATES OF AMERICA

## Abstract

**Background:**

Quantitative muscle water T2 (T2_w_) mapping is regarded as a biomarker for disease activity and response to treatment in neuromuscular diseases (NMD). However, the implementation in clinical settings is limited due to long scanning times and low resolution. Using artificial intelligence (AI) to accelerate MR image acquisition offers a possible solution. Combining compressed sensing and parallel imaging with AI-based reconstruction, known as CSAI (SmartSpeed, Philips Healthcare), allows for the generation of high-quality, weighted MR images in a shorter scan time. However, CSAI has not yet been investigated for quantitative MRI. Therefore, in the present work we assessed the performance of CSAI acceleration for T2_w_ mapping compared to standard acceleration with SENSE.

**Methods:**

T2_w_ mapping of the thigh muscles, based on T2-prepared 3D TSE with SPAIR fat suppression, was performed using standard SENSE (acceleration factor of 2; 04:35 min; SENSE) and CSAI (acceleration factor of 5; 01:57 min; CSAI 5x) in ten patients with facioscapulohumeral muscular dystrophy (FSHD). Subjects were scanned in two consecutive sessions (14 days in between). In each dataset, six regions of interest were placed in three thigh muscles bilaterally. SENSE and CSAI 5x acceleration were compared for i) image quality using apparent signal- and contrast-to-noise ratio (aSNR/aCNR), ii) diagnostic agreement of T2w values, and iii) intra- and inter-session reproducibility.

**Results:**

aSNR and aCNR of SENSE and CSAI 5x scans were not significantly different (*p* >  0.05). An excellent agreement of SENSE and CSAI 5x T2_w_ values was shown (r =  0.99; ICC =  0.992). T2_w_ mapping with both acceleration methods showed excellent, matching intra-method reproducibility.

**Conclusion:**

AI-based acceleration of CS data allows for scan time reduction of more than 50% for T2_w_ mapping in the thigh muscles of NMD patients without compromising quantitative validity.

## Introduction

As active pathological changes, patients with neuromuscular diseases (NMD) show alterations of muscle water content caused by inflammation, denervation, or dystrophy. These precede long-term chronic degeneration, characterized by intramuscular fat accumulation and fibrosis [1–3]. Pathological changes in muscle composition can be noninvasively examined with magnetic resonance imaging (MRI). In the diagnostic workup of NMD patients, currently mainly qualitative, weighted muscle MRI is assessed using subjective, reader-dependent rating scales [4, 5].

Therefore, the development of quantitative MRI techniques has recently been fostered, which promise to provide absolute, objective tissue measurements. Whereas muscle global T2 is a measure of the overall water and fat signal in the muscle tissue, the quantitative magnetic resonance (MR) biomarker muscle water T2 (T2_w_) allows for the determination of the chemically selective muscle water component [3,6]. T2_w_ is highly sensitive but rather unspecific, being influenced by various circumstances such as inflammation, necrosis, denervation, or exercise. Nevertheless, T2_w_ is currently seen as a marker for disease activity, possibly predicting degenerative changes [1,3,7].

However, the simultaneous presence of fatty degeneration and oedematous changes in the same muscle in patients with NMD makes a reliable quantification of T2_w_ challenging. Both factors, water and fat, influence the T2 signal and cannot easily be distinguished from each other. As single voxel ^1^H magnetic resonance spectroscopy (MRS) can resolve and separate the fat and water signal, it is seen as the reference standard for T2_w_ determination, with the drawback of missing spatial resolution. Chemical shift imaging is time-consuming, artefact-prone and the spatial resolution is still limited [8]. To overcome these limitations, quantitative T2_w_ mapping sequences have been developed. Different methods, e.g., the Dixon technique or modelling of the multiexponential T2 decay with or without extended phase graphs (EPG) can be used to determine the chemically selective T2_w_ [9–12]. Another approach, using an adiabatic BIR-4 radiofrequency (RF) pulse in a T2-preparation module in combination with spectral adiabatic inversion recovery (SPAIR) fat suppression and a 3D turbo spin echo (TSE) readout, showed promising results for T2_w_ measurements, with reduced sensitivity to magnetic field inhomogeneities and fatty infiltration [8,13,14]. The sequence enables T2_w_ determination already during acquisition without the need to model the presence of fat, and the measured T2_w_ values showed good agreement with MRS [8,13,14].

Quantitative MRI is time-consuming, sensitive to motion and thus requires motionlessness during the exam. Patients with NMD often have difficulty lying still in distinct positions for prolonged periods of time due to muscle shortening with increasing stiffness [2], loss of muscle strength or myotonia and contractures [15]. Furthermore, patients with a genetic NMD often get diagnosed in childhood, where a short examination time is key. Consequently, to foster the implementation of T2_w_ mapping techniques in the clinical routine, it is essential to accelerate image acquisition to reduce examination times and thus improve patient experience, reduce motion artifacts and examination costs and increase patient throughput.

Over time different methods to accelerate MRI have been developed. Parallel imaging (PI), known as SENSE or GRAPPA, compressed sensing, or the combination of both are conventional methods, which are already established in clinical routine, to accelerate MR image acquisition. Recently, deep learning (DL) based methods such as convolutional neuronal networks (CNNs) integrated into the image-reconstruction and post-processing steps or generative adversarial networks (GANs) [16–19] have become increasingly promising to accelerate the MRI process. To this end combination of a DL-based reconstruction framework with compressed sensing and parallel imaging using SENSE (CSAI) has been shown to produce high-quality, weighted images while reducing acquisition times, e.g., of the ankle, shoulder, spine, coronary arteries or prostate [20–24].

Although the potential impact of DL-based reconstruction frameworks on quantitative data is of high interest, DL-based acceleration methods for quantitative MRI are still rarely studied [25, 26]. Especially the CSAI approach has not yet been applied to T2_w_ mapping data of NMD patients using the T2-prepared 3D TSE SPAIR sequence.

Therefore, we evaluated the performance of CSAI-based image acceleration for T2_w_ mapping in the thigh musculature of NMD patients using the T2-prepared 3D TSE with SPAIR. The hypothesis was that quantitative precision could be maintained while reducing the scan time by more than half using CSAI with an acceleration factor of 5 (CSAI 5x) compared to conventional acceleration used in the past. To this end, we compared the T2_w_ maps acquired and reconstructed using conventional SENSE versus the novel CSAI acceleration technique in terms of (i) image quality, (ii) diagnostic agreement, and (iii) reproducibility.

## Materials and methods

### Subjects

Ten patients (5 women; mean age 49.7 years (range: 33-64 years), bodyweight mean 76.9 kg (range: 61–98 kg), height mean 182.2 cm (range: 169–198 cm), BMI mean 23.1 (range: 19–27) with facioscapulohumeral muscular dystrophy (FSHD) were recruited between July 19, 2021 and November 27, 2021. The diagnosis was based on genetic testing. The study was approved by the local institutional Committee on Human Research of the Klinikum rechts der Isar, Technical University of Munich (TUM). In line with the local ethics guidelines and participant privacy policies, data cannot be shared publicly. Acquired data will be shared upon reasonable request and based on a formal data sharing agreement. All patients provided written informed consent prior to study participation.

### Data acquisition

The patients’ bilateral thigh region was scanned in a head-first supine position with a 3 T MR system (Ingenia Elition X, Philips Healthcare, Best, The Netherlands). A built-in posterior coil with 12 channels in combination with an anterior coil with 16 channels placed on top of the hip and thigh region were used for signal reception. The images were acquired as consecutive axial stacks covering the bilateral midthigh region on both sides, with the central axial slice of the field of view (FOV) exactly in the middle between the femur head and tibia condyles. The scan protocol included a T2-weighted 2D TSE sequence for qualitative assessment of the muscles and T2_w_ mapping employing a T2-prepared 3D TSE with SPAIR [8,13,14]. The T2_w_ mapping sequence was obtained two times: (i) with an acceleration factor of 2 using SENSE (standard acceleration factor as presented in Weidlich et al. [14]) and (ii) with an acceleration factor of 5 using CSAI (CSAI 5x). While the SENSE acquisition was based on a coherent regular undersampling pattern in k-space, the CSAI acquisition was based on a pseudo random, density weighted, incoherent k-space sampling with higher sampling density in the central part and continuously decreasing density towards the outer parts of k-space. The scan duration of the T2_w_ mapping sequence with SENSE acceleration was 04:35 min and with CSAI 5x reconstruction was 01:57 min ( > 50% shorter compared to SENSE). Detailed sequence parameters are listed in Table 1.

**Table 1:** Sequence parameters of T2-weighted 2D Dixon and SPAIR T2-prepared 3D TSE with SENSE and CSAI 5x acceleration. TR, repetition time; TE, echo time; ms, milliseconds; AP, Anterior-posterior; RL, right-left; RF, radio frequency; TSE, turbo spin echo; FOV, field of view; min, minutes; SPAIR, spectral adiabatic inversion recovery; FH: feet-head.

Sequence	T2w 2D Dixon TSE	SENSE T2-prepared 3D TSE with SPAIR	CSAI 5x T2-prepared 3D TSE with SPAIR
TR [ms]	7000	1500	1500
TE [ms]	100	18	18
Δ TE [ms]	1.0	–	–
Number of slices	26	30	30
Slice gap [mm]	6	0	0
Acquisition voxel (AP x RL x slice thickness [mm³]	2.2 x 2.18 x 6.0	2 x 2 x 8	2 x 2 x 8
T2 preparation duration [ms]	–	20/30/40/50/60	20/30/40/50/60
RF-pulses (in readout)	–	Flip angle modulated TSE readout (echo spacing: 2.4 ms, 5 start-up echoes)	Flip angle modulated TSE readout (echo spacing: 2.4 ms, 5 start-up echoes)
TSE factor	21	50	50
Averages	2	Partial averaging (1.4)	Partial averaging (1.4)
FOV [mm³]	330 x 453 x 306	420 x 262 x 120	420 x 262 x 120
Acceleration factor	CS-SENSE with a reduction factor of 3	SENSE with a reduction factor of 2 in AP direction and factor of 1 in FH direction	CSAI with a reduction factor of 5, denoising strong
Scan time [min]	01:31 min	4:35 min	1:57 min

**Fig 1:**
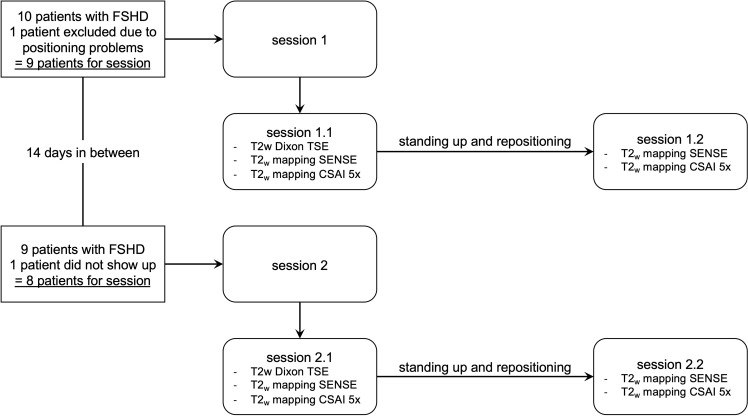
Flow-chart of the scanning procedure. FSHD, facioscapulohumeral muscular dystrophy; T2w, T2 weighted; TSE, turbo spin echo; T2_w_, muscle water T2.

Scanning was performed in two consecutive sessions with 14 days in between, referred to as session 1 and session 2. During each session the midthigh region of each patient was scanned first with the T2-weighted Dixon TSE sequence, followed by T2_w_ mapping with SENSE and CSAI 5x, respectively (session 1.1/2.1). After standing up and repositioning, T2_w_ mapping was performed again with SENSE and CSAI 5x, respectively (session 1.2/2.2). The order of SENSE and CSAI 5x T2_w_ mapping was randomly changed to reduce confounding effects such as increased motion with scan time. Patients were asked to have the same physical load before session 1 and 2. Particularly, there were instructed to maintain a similar level of physical activity on the day of scanning, which included walking approximately the same distance and number of stairs, avoiding or performing the same amount of exercise, and adhering to similar routines as much as possible prior to session 1 and 2. The complete scanning procedure is shown in Fig 1. One patient was excluded during session 1 due to positioning problems caused by the disease. One patient did not show up for session 2 and therefore was excluded from any comparison studies between sessions 1 and 2.

### Data reconstruction

The T2-weighted 2D Dixon TSE was processed online using the vendor’s mDixon algorithm [27, 28]. Reconstruction of CSAI accelerated T2-prepared 3D TSE with SPAIR raw image data was based on the Adaptive-CS-Net presented by Pezzotti et al. [29], implemented by the vendor (SmartSpeed, Philips Healthcare, Best, The Netherlands) and has been described previously [20–24]. In short, CSAI effectively combines PI using SENSE and compressed sensing with a CNN-based DL approach. In this approach the CNN replaces the sparsifying transform in the combined iterative SENSE and compressed sensing reconstruction, which ensures data consistency and incorporates prior knowledge such as coil sensitivity distribution and location of the image background. CSAI reconstruction of raw images was done online directly at the scanner with reconstruction times of about 1 minute.

Estimation of quantitative T2_w_ maps from the raw image data was performed offline using Matlab (MathWorks, Natick, MA, USA; version R2017b). An exponential fit with linear least-squares was used to fit the data described by the following equation:


$$S=S_{0}*e^{-\frac{T2prep}{T2}}$$ (1)

without fitting of the baseline. Magnitude data were used.

### Qualitative pathology rating

The muscles were rated using the qualitative T2-weighted Dixon TSE sequence for fatty infiltration in the fat-only image and for oedematous changes in the water-only image by one neuroradiologist with two years of experience. Fatty infiltration was rated according to the Mercuri grading scale [4], and oedematous alterations according to the Morrow grading scale [5].

### Assessment of image quality

Image quality of SENSE and CSAI 5x T2_w_ mapping was assessed by visual inspection of the first and last echo of the T2_w_ mapping raw image data, respectively, by two neuroadiologists (two and seven years of experience in muscle imaging).

The neuroradiologist with two years of experience in muscle imaging performed apparent signal- and contrast-to-noise ratio (aSNR/aCNR) measurements comparable to Pennig et al. [30] on the last echo of the T2-prepared 3D TSE with SPAIR raw image data, which exhibits the most sensitivity to noise, with SENSE and CSAI 5x acceleration, respectively.

Two-dimensional ten-pixel circular regions of interest (ROIs) were placed bilaterally in the biceps femoris caput longum in the central slice of the FOV. As a reference standard for background noise, another ROI was placed in the middle of the bone marrow of the femur in the same slice. The ROI values of the right and left side were averaged, respectively. aSNR and aCNR were calculated as follows:


$$aSNR=\frac{SI_{muscle}}{SDofSI_{bonemarrow}}$$ (2)

$$aCNR=\frac{(SI_{muscle}-SI_{bonemarrow)}}{SDofSI_{bonemarrow}}$$ (3)

where *SI* is the signal intensity and *SD* is the standard deviation. For each T2_w_ mapping data set of session 1.1, aSNR and aCNR were calculated. As the scan time of the CSAI 5x scan is significantly reduced compared to the SENSE scan, additionally aSNR and aCNR efficiency (aSNR_eff_ and aCNR_eff_) of CSAI 5x and SENSE were calculated as follows:


$$aSNR_{eff}=\frac{aSNR}{t}\times 100$$ (4)

$$aCNR_{eff}=\frac{aCNR}{t}\times 100$$ (5)

where *t* is the scan time in seconds (275 sec for SENSE; 117 sec for CSAI 5x).

### Analysis of quantitative T2_w_ mapping data

Using ITKSnap [31], ROIs were manually drawn in the first echo of the T2-prepared 3D TSE with SPAIR raw image data with SENSE acceleration of session 1.1 in the following muscles bilaterally: gracilis, biceps femoris caput longum and vastus lateralis. The ROIs were placed in five consecutive axial slices at the midthigh region using a 3D ten-pixel circular brush. The ROIs were transferred and manually adjusted to the same localization in the T2-prepared 3D TSE with SPAIR raw image data of the CSAI acceleration and the subsequent sessions 1.2, 2.1, and 2.2. If any change in localization occurred due to intra- and inter-session movement, the ROI was corrected pixel-accurately to the localization of the ROI of session 1.1. The ROI determination was done by one neuroradiologist with two years of experience in muscle imaging, who ensured to exclude intramuscular vessels, intramuscular septs or similar structures, to merely represent muscle tissue. T2_w_ values of the T2_w_ maps with SENSE versus CSAI 5x were extracted, enabling a quantitative comparison of the two acceleration methods.

### Statistical analysis

The statistical analysis and plot generation was performed using IBM SPSS, version 29.0 (IBM Corp. Released 2022. IBM SPSS Statistics for Windows, Version 29.0. Armonk, NY: IBM Corp). The level of significance (α) was set to *p* <  0.05.

For comparison of T2_w_ mapping with SENSE versus CSAI 5x acceleration, the SENSE acceleration was used as the standard of reference since it is currently the standard acceleration method when performing the T2-prepared 3D TSE with SPAIR [8,13,14].

A Wilcoxon-signed rank test was performed to compare aSNR and aCNR as well as aSNR_eff_ and aCNR_eff_ between SENSE and CSAI 5x T2-prepared 3D TSE with SPAIR raw image data.

To determine the relationship between the T2_w_ values of SENSE and CSAI 5x a two sided Pearson correlation coefficient r was calculated [32].

For analysing the agreement and correlation between SENSE T2_w_ mapping and CSAI T2_w_ mapping, the intraclass correlation coefficient (ICC) was calculated based on a mean rating (k =  2), absolute agreement, two-way mixed-effect model with average measures [33]. The ICC was calculated for general agreement and subdivided for pathology and spatial relation. According to [33], ICC values less than 0.5 indicate poor reliability, values between 0.5 and 0.75 indicate moderate reliability, values between 0.75 and 0.9 indicate good reliability, and values greater than 0.90 indicate excellent agreement.

For the quantitative T2_w_ value comparison between SENSE and CSAI 5x acceleration of T2_w_ mapping, a Bland-Altman analysis was conducted, using the true value varies method as described in [34–36]. As a prerequisite the difference between T2_w_ values of SENSE and CSAI 5x acceleration of T2_w_ mapping was tested for normal distribution using histograms and statistically with the Kolmogorov-Smirnov-Test and the Shapiro-Wilk-Test. The bias and the upper and lower limit of agreement (LoA) with their 95% confidence intervals (CI) were calculated and plotted; the within subject variance (WSV), the between subject variance (BSV) with their standard errors (SE), the spearman correlation coefficient ρ and the repeatability coefficients were calculated [34–36].

## Results

### Image quality

**Fig 2:**
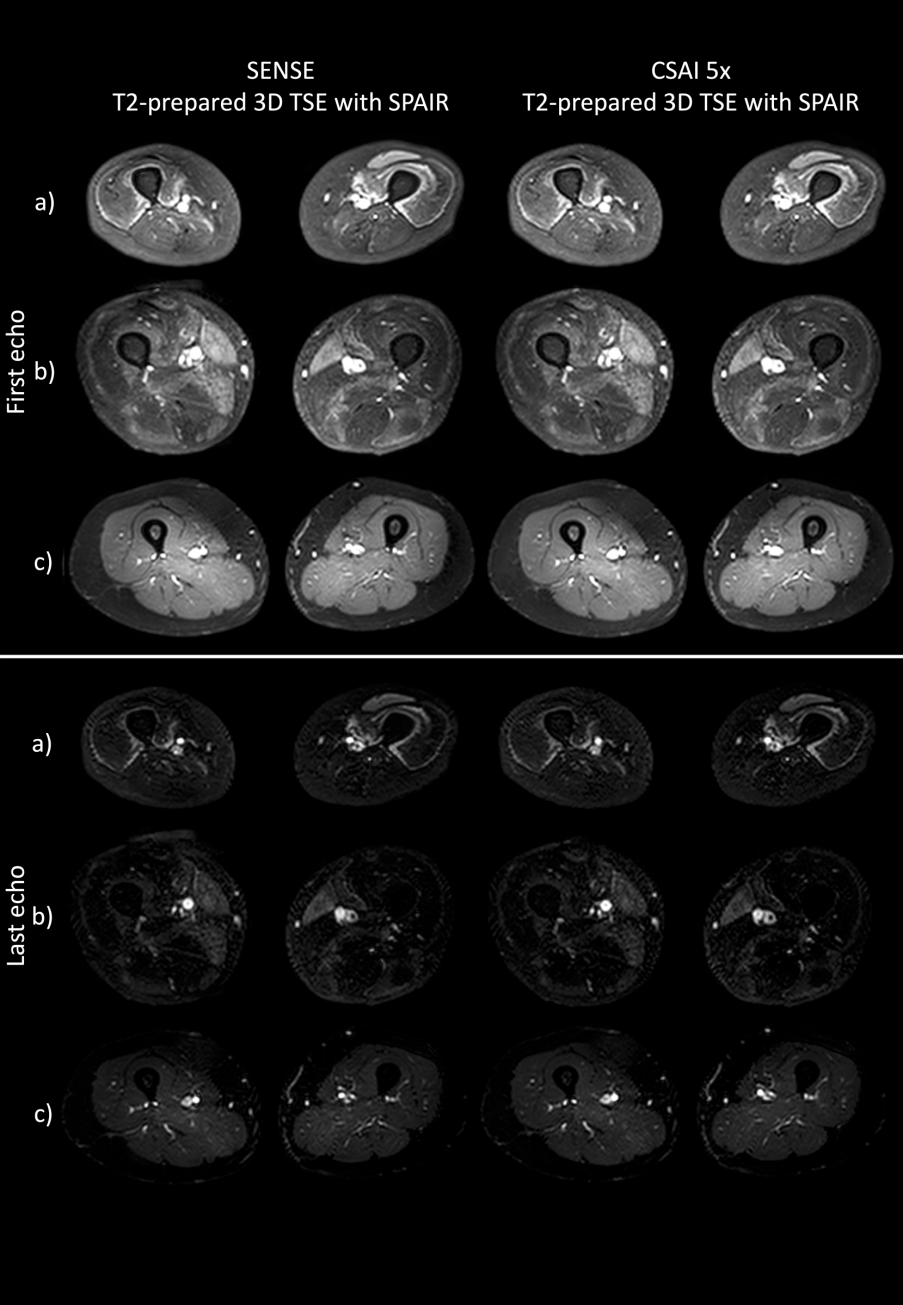
Representative T2-prepared 3D TSE with SPAIR raw image data of first echo (top three rows) and last echo (lower three rows) of three patients (a, b, c) with standard SENSE (left) and CSAI 5x (right) acceleration. TSE, turbo spin echo; SPAIR, spectral adiabatic inversion recovery.

As shown in Fig 2, a visual inspection of the first and last echo of the T2_w_ mapping raw image data revealed no difference in image quality of SENSE versus CSAI 5x T2_w_ mapping. The objective assessment of the image quality of the T2-prepared 3D TSE with SPAIR raw image data using aSNR and aCNR did not show any significant difference (*p* >  0.05) between SENSE and CSAI 5x with slightly lower values for the CSAI 5x scan than for the SENSE scan. When normalized with scan time aSNR_eff_ values were significantly higher for CSAI 5x T2_w_ mapping versus SENSE T2_w_ mapping (*p* =  0.027), while aCNR_eff_ showed a clear tendency towards higher values for CSAI 5x T2_w_ mapping (*p* >  0.05). The detailed values are provided in S1 Table.

### Diagnostic agreement

**Fig 3:**
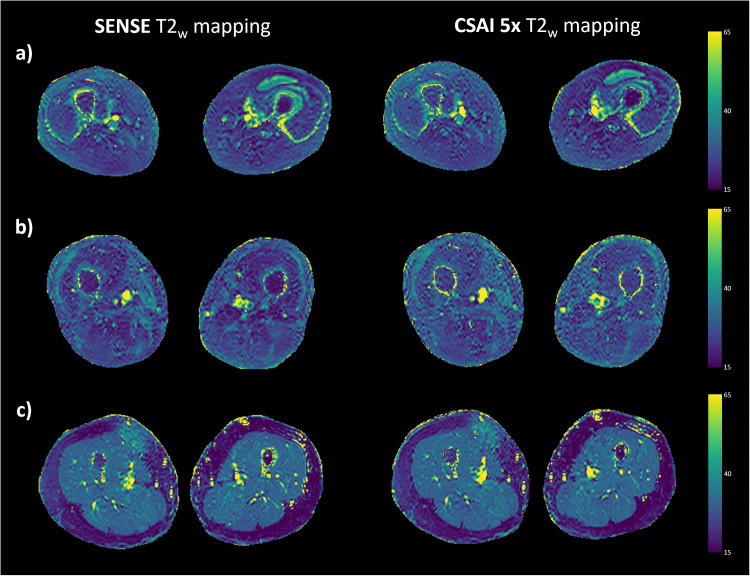
Exemplary T2_w_ maps from three representative patients (a, b, c) with SENSE and CSAI 5x acceleration, colorbar on the right.

In Fig 3 exemplary T2_w_ maps are shown, in which the ROI-based measurements were conducted on both sides in the muscles: biceps femoris caput longum, gracilis and vastus lateralis.

**Fig 4:**
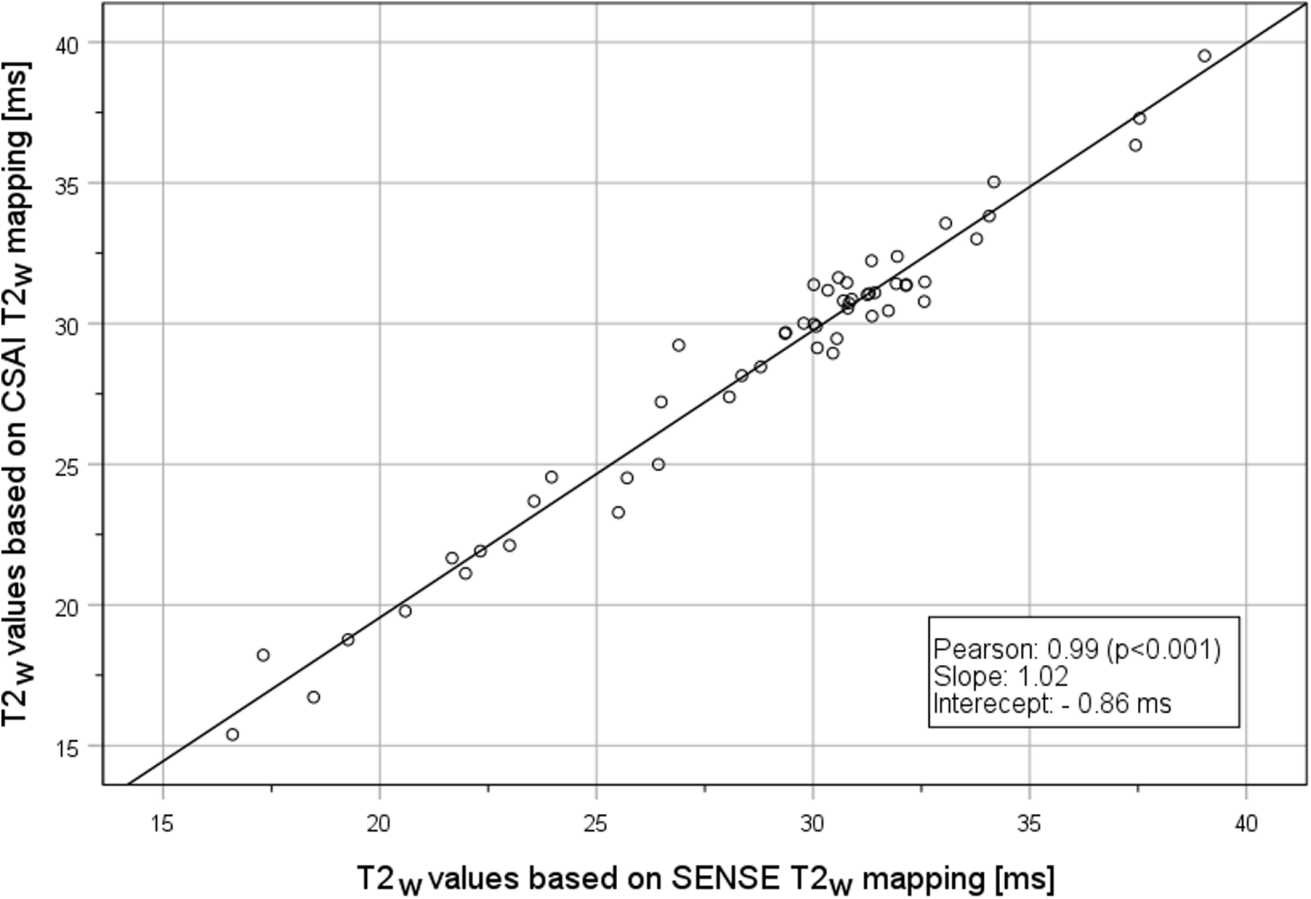
Correlation analysis of T2_w_ values from T2_w_ mapping with SENSE versus T2_w_ mapping with CSAI 5x from session 1.1.

An overall excellent agreement between the quantitative T2_w_ values based on SENSE versus the CSAI 5x T2_w_ mapping of session 1.1. was reported (r =  0.99; *p* <  0.001; slope =  1.02; intercept =  -0.86 ms) (Fig 4). The ICC for SENSE versus CSAI 5x T2_w_ mapping was 0.992 (95% CI: 0.985 – 0.995; *p* <  0.05).

**Fig 5:**
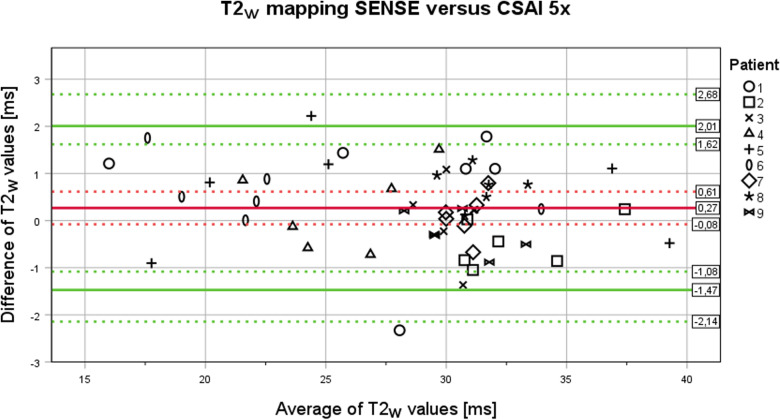
Bland-Altman plot of T2_w_ values from T2_w_ mapping with SENSE versus T2_w_ mapping with CSAI 5x from session 1.1. Six measurements were obtained from each of the nine patients, respectively. Bias (0.27 ms) is indicated as a red line, upper (2.01 ms) and lower (-1.47 ms) limits of agreement are indicated as green lines, and the corresponding 95% confidence interval are shown as broad dashed lines in the corresponding color.

Based on a Bland-Altman analysis of the T2_w_ values from session 1.1 (Fig 5) the bias of the differences was 0.27 ms (95% CI: -0.08 ms – 0.61 ms), the upper LoA was 2.01 ms (95% CI: 1.62 ms – 2.68 ms), the lower LoA was -1.47 ms (95% CI: -1.08 ms – -2.14 ms), the WSV was 0.7019 (SE + /- 0.1480), and the BSV was 0.0860 (SE: + /- 0.1044). The Spearman rank correlation coefficient showed no significant correlation between the differences and the mean, thus there was no trend in the bias over the measured values. Inter- and intra-subject effects could be excluded as influential factors. The repeatability coefficient of CSAI 5x T2_w_ mapping with 4.40 ms (95% CI: 3.65 ms – 5.54 ms) was very similar to the repeatability coefficient of SENSE T2_w_ mapping with 4.36 ms (95% CI: 3.62 ms – 5.49 ms) with a ratio of 0.99 (95% CI: 0.91 – 1.08). Of note, the absolute values of the repeatability coefficients are not directly applicable, as no separation for the underlying pathology (healthy, oedematous, or fatty infiltrated muscles) was performed.

A subgroup analysis showed excellent correlation between SENSE and CSAI 5x T2_w_ mapping particularly in muscles with severe pathological alterations. The ICC for SENSE versus CSAI 5x T2_w_ mapping in highly oedematouse muscles (Morrow grade 2) was 0.988 (95% CI: 0.966 – 0.996) and in highly fatty infiltrated muscles (Mercuri grade 4) was 0.980 (95% CI: 0.927 – 0.995).

A second subgroup analysis showed excellent correlation between SENSE and CSAI 5x T2_w_ mapping also when the investigated muscles were considered separately. The ICC for SENSE versus CSAI 5x T2_w_ mapping was 0.968 (95% CI: 0.804 – 0.991) for the gracilis, 0.996 (95% CI: 0.988 – 0.998) for the biceps femoris caput longum, and 0.993 (95% CI: 0.983 – 0.997) for the vastus lateralis.

### Reproducibility

T2_w_ mapping with both acceleration methods showed excellent intra-method reproducibility. The ICC values for SENSE and CSAI T2_w_ mapping within session 1 and 2 (intra-session intra-method reproducibility) showed no relevant differences between both acceleration methods. The ICC values for SENSE and CSAI 5x T2_w_ mapping between session 1 and 2 (inter-session intra-method reproducibility) were slightly lower, however still excellent, and again with no relevant differences between both acceleration methods. Detailed ICC values and corresponding 95% CI are shown in Table 2.

**Table 2:** ICC for intra- and inter-session reproducibility of T2_w_ mapping with SENSE and CSAI 5x. ICC, intraclass correlation coefficient; CI, confidence interval.

	Intra-session intra-method reproducibility		Inter-session intra-method reproducibility	
Session	1.1 vs. 1.2	2.1 vs. 2.2	1.1 vs. 2.1	1.2 vs. 2.2
SENSE T2_w_ mapping ICC (95% CI)	0.987 (0.976–0.992)	0.984 (0.965–0.992)	0.976 (0.958–0.987)	0.984 (0.971–0.991)
CSAI 5x T2_w_ mapping ICC (95% CI)	0.988 (0.974–0.994)	0.989 (0.971–0.995)	0.982 (0.968–0.990)	0.979 (0.962–0.988)

## Discussion

In this prospective study, we investigated the performance of a novel DL-based image acceleration approach (CSAI) for quantitative imaging. Compared to the standard acceleration with SENSE, CSAI allowed for a scan time reduction of more than 50% for T2_w_ mapping based on the T2-prepared 3D TSE with SPAIR fat suppression, while showing excellent agreement of T2_w_ values and comparable intra-method reproducibility.

T2_w_ is currently seen as a promising MR biomarker for assessment of disease activity and therapy response in patients with NMD [1,3,7,37]. However, T2_w_ mapping is still mainly performed in research studies, while an implementation in the clinical routine is missing not least because of limited examination times per patient [38]. For the translation of quantitative MR techniques from research to clinical routine scan time reduction is crucial. The presented DL-based acceleration of T2_w_ mapping might foster the implementation of T2_w_ mapping in the clinical routine and consequently enhance objective evaluation of muscle pathologies in NMD patients.

Previous studies investigated the performance of the CSAI acceleration approach in a variety of anatomical regions such as the ankle, spine, coronary arteries, and prostate allowing for a significant reduction of acquisition times without decreasing diagnostic image quality [20,22–24]. While previous studies investigated its potential for qualitative imaging, to the best of the authors’ knowledge the present work is the first in which CSAI is used for quantitative imaging.

While aSNR and aCNR of the CSAI 5x scan compared to the SENSE scan were slightly trending lower, they showed no significant difference, highlighting that the applied acceleration factor of 5 is feasible and still providing sufficient signal for the presented setup. Of note, when normalized for the scan time aSNR_eff_ values for the CSAI 5x scan were significantly higher than for the SENSE scan, while aCNR_eff_ values for the CSAI 5x scan showed a clear tendency towards higher values for the CSAI 5x scan. The aim of the present pilot study was to investigate whether acceleration of quantitative imaging with CSAI is possible. The use of aSNR and aCNR as a measure to determine the perfect threshold for the acceleration factor that allows the fastest possible imaging while maintaining sufficient image quality will be part of future research.

Quantitative ROI measurements in the T2_w_ maps showed perfect agreement of T2_w_ values with CSAI 5x and SENSE acceleration. Consequently, the diagnostic performance of T2_w_ mapping based on the T2-prepared 3D TSE with SPAIR fat suppression using the present CSAI 5x acceleration can be considered equivalent to SENSE.

Of note, the excellent agreement between the T2_w_ values of SENSE and CSAI 5x was independent of i) the muscle studied and ii) the underlying pathology: high ICCs were observed for all investigated muscles (gracilis, biceps femoris caput longum, and vastus lateralis) and for both, severely edematous muscles and severely fat-infiltrated muscles. T2_w_ mapping based on the T2-prepared 3D TSE with SPAIR was shown to be relatively insensitive to B0 and B1 inhomogeneities as well as to fatty infiltration [8,13,14]. The present work underlines that the inherent advantages of the sequence also hold true when the CSAI 5x image acceleration approach is applied.

However, as shown in the work by Schlaeger et al. [39], particularly in heterogeneously affected muscle tissue of NMD patients, where edematous and fatty changes may be present at the same time, a mere mean value analysis is questionable and a deeper analysis of the underlying T2_w_ value distribution is required. Such histogram analysis and corresponding agreement of the T2_w_ value distribution between SENSE and CSAI 5x T2_w_ mapping will be part of further studies.

Both acceleration methods showed excellent intra-session (during the same session after repositioning) and inter-session (14 days between two sessions) reproducibility, with no relevant differences between SENSE and CSAI 5x T2_w_ mapping. Intra-session reproducibility was within the range of current literature [40]. Inter-session reproducibility was slightly worse than intra-session reproducibility. Because T2_w_ is a rather unspecific MR biomarker that is influenced by a variety of circumstances such as inflammation, necrosis, denervation, or exercise [40–42], e.g., minimal alteration in exercise level prior to the study already might have a major impact on the measured T2_w_ values. Although patients were asked to experience the same level of physical exertion before sessions 1 and 2 to control the actual amount of exercise intensity remains challenging, potentially resulting in a slightly lower ICC for inter-session reproducibility.

## Limitations

The present study is not without limitations. First, no external standard of reference as corresponding muscle biopsy results were available to serve as a ground truth for the measured pathological changes in the muscle tissue. However, the T2-prepared 3D TSE T2_w_ with SPAIR sequence has been validated before with MRS [8]. Second, the total of nine included patients was rather small. However, the present study was performed in patients with rare diseases, where results in a small cohort can already add significant value to the diagnostic workup. Particularly, the simultaneously present edematous and fatty changes in muscle tissue of these patients pose a challenge for T2_w_ mapping techniques, making NMD patients a perfect cohort for testing the techniques. Third, the study population was restricted to one specific NMD (FSHD), so the generalizability to other NMD must be investigated. Furthermore, as this study’s purpose was a validation study conducted on an internal dataset, additional studies should include multicentric data to verify external validity. Fourth, to confirm the clinical value of T2_w_ as an imaging biomarker, studies with correlation of quantitative imaging and clinical measurements, e.g., laboratory results, walking distance, symptom severity and therapy effectiveness are needed. For this purpose, correlation studies with clinical parameters and longitudinal assessment of T2_w_ values with the present sequence will be part of further research. Fifth, the aim of this pilot study was to investigate whether acceleration of T2_w_ mapping based on the T2-prepared 3D TSE with SPAIR using CSAI is feasible. The results demonstrate that the applied acceleration factor of 5 is both robust and reproducible in providing T2_w_ values. The findings suggest that even higher acceleration factors or higher resolution with the same scan time might be achievable using CSAI. These possibilities will be explored in future research.

## Conclusion

DL-based acceleration of CS data allows for scan time reduction of more than 50% for T2_w_ mapping in the thigh muscles of NMD patients without compromising quantitative validity. CSAI allowed for a significant scan time reduction for T2_w_ mapping based on the T2-prepared 3D TSE with SPAIR fat suppression, while showing excellent agreement of T2_w_ values and comparable intra-method reproducibility. DL-based acceleration of MR image acquisition can facilitate the translation of quantitative MR techniques from research to clinical practice, enhancing the objective evaluation of muscle pathologies in NMD patients.

## Supporting information

Table S1 aSNR, aSNReff, aCNR and aCNReff values of the SENSE scan versus the CSAI 5x scan. aSNR and aCNR were not significantly different between SENSE and CSAI 5x T2w mapping (p >  0.05), while aSNReff was significantly higher for the CSAI 5x scan compared to SENSE scan (p =  0.027). aCNReff values of the CSAI 5x scan showed a clear tendency towards higher values for the CSAI 5x scan (p >  0.05). (DOCX)
