# Supplementary material for: Deep learning-based acceleration of muscle water T2 mapping in patients with neuromuscular diseases by more than 50% - translating quantitative MRI from research to clinical routine
Source: PLoS One. 2025 Apr 16;20(4):e0318599. doi: 10.1371/journal.pone.0318599 (PMC12002432; doi:10.1371/journal.pone.0318599)
Supplement: Table S1 — (DOCX) [file pone.0318599.s001.docx]

**Supplementary Material**

|  | **aSNR** | | **aSNR_eff_** | | **aCNR** | | **aCNR_eff_** | |
| --- | --- | --- | --- | --- | --- | --- | --- | --- |
| **Patient** | **SENSE** | **CSAI 5x** | **SENSE** | **CSAI 5x** | **SENSE** | **CSAI 5x** | **SENSE** | **CSAI 5x** |
| 1 | 27.8 | 11.0 | 10.1 | 9.4 | 24.3 | 8.7 | 8.8 | 7.4 |
| 2 | 17.7 | 16.7 | 6.4 | 14.2 | 15.7 | 14.7 | 5.7 | 12.5 |
| 3 | 11.5 | 8.1 | 4.2 | 6.9 | 9.5 | 6.4 | 3.5 | 5.4 |
| 4 | 4.7 | 2.5 | 1.7 | 2.1 | 1.9 | 0.8 | 0.7 | 0.7 |
| 5 | 2.5 | 3.0 | 0.9 | 2.6 | 0.2 | 1.3 | 0.1 | 1.1 |
| 6 | 3.1 | 1.3 | 1.1 | 1.1 | 1.3 | -0.2 | 0.5 | -0.1 |
| 7 | 3.4 | 2.8 | 1.2 | 2.4 | 2.3 | 1.7 | 0.8 | 1.4 |
| 9 | 12.0 | 15.9 | 4.4 | 13.6 | 10.3 | 13.9 | 3.8 | 11.9 |
| 10 | 13.3 | 12.2 | 4.8 | 10.4 | 11.6 | 9.9 | 4.2 | 8.5 |
| Mean ± SD | 10.7 ± 7.9 | 8.2 ± 5.7 | 3.9 ± 2.9 | 7.0 ± 4.9 | 8.6 ± 7.6 | 6.3 ± 5.4 | 3.1 ± 2.7 | 5.4 ± 4.6 |

Table S1: aSNR, aSNR_eff_, aCNR and aCNR_eff_ values of the SENSE scan versus the CSAI 5x scan. aSNR and aCNR were not significantly different between SENSE and CSAI 5x T2_w_ mapping (p > 0.05), while aSNR_eff_ was significantly higher for the CSAI 5x scan compared to SENSE scan (p = 0.027). aCNR_eff_ values of the CSAI 5x scan showed a clear tendency towards higher values for the CSAI 5x scan (p > 0.05).
